# Older Patients’ Competence, Preferences, and Attitudes Toward Digital Technology Use: Explorative Study

**DOI:** 10.2196/27005

**Published:** 2021-05-14

**Authors:** Rikke Terp, Lars Kayser, Tove Lindhardt

**Affiliations:** 1 Department of Internal Medicine Herlev and Gentofte Hospital Copenhagen University Hospital Hellerup Denmark; 2 Department of Public Health University of Copenhagen Copenhagen Denmark

**Keywords:** eHealth literacy, eHealth, self-management, older patients, explorative study

## Abstract

**Background:**

Malnutrition is prevalent in older patients, which is associated with severe consequences such as a decline in functional status, increased risk of readmission, and increased mortality. A tablet-based eHealth solution (Food‘n’Go) was recently developed and introduced at our clinic to support older patients’ involvement in nutritional interventions during their hospitalization, thereby enhancing their awareness and motivation for choosing the right food to obtain sufficient calorie and protein intake. To reap the full benefits from the eHealth solution, the technology should be introduced and accompanied by support that targets the end users’ competence level and needs.

**Objective:**

In this study, we aimed to explore older patients’ readiness (ie, competence, preferences, and attitudes) toward the use of information and communication technology (ICT), and to identify the factors that may act as barriers or facilitators for their engagement with health technology.

**Methods:**

A descriptive and explorative study was performed using triangulation of data derived from semistructured interviews and questionnaires (based on the Readiness and Enablement Index for Health Technology [READHY] instrument). Older hospitalized patients (age ≥65 years; N=25) were included from two hospitals in Denmark.

**Results:**

The majority (16/25, 64%) of the older patients (median age 81 years) were users of ICT. The qualitative findings revealed that their experiences of benefits related to the use of ICT facilitated usage. Barriers for use of ICT were health-related challenges, limited digital literacy, and low self-efficacy related to ICT use due to age-related prejudices by their relatives and themselves. The qualitative findings were also reflected in the low median scores on the eHealth Literacy Questionnaire (eHLQ) READHY scales within dimensions addressing the user’s knowledge and skills (eHLQ1:1.8; eHLQ3: 2.0), and the user experience (eHLQ6: 2.0; eHLQ7: 1.5).

**Conclusions:**

Older patients are potential users of ICT, but experience a variety of barriers for using eHealth. When introducing older patients to eHealth, it is important to emphasize the possible benefits, and to offer support targeting their knowledge, skills, and motivation.

## Introduction

Malnutrition is a prevalent and challenging area in health care for older patients [1-3] with severe consequences such as decreased physical function [1], prolonged hospitalization [4], readmissions [5], and mortality [1,4,6]. Multiple interventions targeting the prevention of malnutrition in older patients have been investigated, and the majority consist of dietary interventions with varying effects [7-9]. To support older patients in eating adequately, interventions that address the individual’s motivation and preferences are required [10]. Hence, patient involvement is a prerequisite, and eHealth technology may be a useful tool in this regard. However, few technology studies have focused on the management of malnutrition, and only a limited number of such studies have included older patients. It is commonly considered that older patients do not utilize and benefit from digital technologies [11-13]. Due to this faulty assumption, older people are given less opportunities to use eHealth [13,14]. Indeed, former studies have described a positive attitude among older patients toward digital technologies [15-18], but that they may have less experience with these tools than younger people [19]. These results are supported by data from Statistics Denmark, which show a steady increase in the use of digital technologies among older age groups; in 2019, 85% of people aged 75 to 89 years used internet banking compared to only 61% in 2011 [20]. Former studies have investigated the specific barriers for older patients to use digital technologies [11,16,17,21], identifying lack of digital literacy, knowledge, and confidence in using technology as predominant barriers. However, this is a new and expanding research area and the evidence remains limited. Moreover, an understanding of older patients’ capacity to engage with digital technologies requires insight into their knowledge, skills, and perception of the technology (ie, eHealth literacy) [22], taking the social context into consideration [23]. Recently the Readiness and Enablement Index for Health Technology (READHY) instrument was developed, which can capture not only individuals’ eHealth literacy but also the social context, and their ability to manage the burden of treatment and illness [24]. 

In a recent project implemented at two hospitals in the Copenhagen area, our research group, in collaboration with the information technology company Movesca, developed a new eHealth solution (Food‘n’Go) with the aim of supporting older patients (>65 years) to participate in nutritional interventions while hospitalized, thereby enhancing their awareness and motivation for eating sufficiently [25]. Food‘n’Go is an app provided on a computer tablet where the patients can (1) access a menu of food choices, (2) order meals, (3) register food intake, and (4) receive feedback. To reap the full benefits from such an eHealth solution, it should be introduced and accompanied by support targeted to the end user’s competence and needs. Therefore, as an adjunct study to the above technology study, we are developing an educative intervention supporting older patients in their use of this eHealth tool to increase the adoption and advantages of using the technology. Development of such an educative intervention requires not only knowledge of the end user’s competencies, needs, and abilities to participate in the nutritional interventions but also to address the readiness for usage of technology.

Toward this end, the aim of this study was to explore older patients’ competencies, preferences, and attitudes toward use of information and communication technology (ICT), and to gain an understanding of the barriers and facilitators for their motivation to engage with eHealth.

## Methods

### Design

The overall design has been reported elsewhere [26]. Briefly, this report builds on field studies that addressed older patients’ competencies, preferences, and attitudes toward food and technology. The focus on nutrition and food has been reported previously [26]. We here report our findings in relation to the technology perspective. In short, we recapture the principles of the study design to establish the context for the results, analysis, and discussion. This study applied a descriptive and explorative design using data triangulation. Table 1 illustrates the methodology for inclusion, recruitment, data collection, and analysis.

**Table 1:** Description of participant recruitment, inclusion and exclusion criteria, data collection, and data analysis.

Stage of the study				Description
**Recruitment: consecutive sampling**				
	Inclusion criteria		Age ≥65 years (N=25) Admitted at one of the two selected hospital units specialized in internal medicine: Hospital A (n=12) and Hospital B (n=13)	
	Exclusion criteria		Total excluded N=60 Already included (n=6, 10%), unwilling to participate (n=12, 20%), terminal illness (n=2, 3%), discharged before inclusion (n=13, 22%), unable to provide informed consent (n=27, 45%)	
**Data collection**				
	Time period		March 2017 to July 2017^a^	
	Interviews		Individual semistructured interviews, interview guided by READHY^b^ dimensions	
**Data analysis**				
	Qualitative data		Content analysis; coded with an inductive approach using the management software program NVivo 11	
	**Quantitative data**			
		Descriptive statistics	READHY scores, participant characteristics	
		Test statistics	χ^2^ (categorical variables), Mann-Whitney *U* test (continuous variables); *P*<.05 indicated significance analyzed with SPSS version 25	

^a^Except for two male participants who were included in March 2018 due to overrepresentation of women.

^b^READHY: Readiness and Enablement Index for Health Technology.

### Participants and Procedure

The participants (25 hospitalized patients) were recruited from two units specialized in internal medicine from two hospitals under the same administration in Denmark. To capture as much variation as possible in competencies, preferences, and attitudes toward ICT in the group of older patients, we consecutively included the participants using a cross-sectional sampling strategy. On randomly selected days, patients fulfilling the inclusion criteria were included. To ensure heterogeneity in terms of socioeconomic status, we purposefully included participants from two different hospital units. The two hospitals (Hospital A and Hospital B) serve different populations regarding socioeconomic status. People living in the uptake area of Hospital B have a lower socioeconomic status compared to those in the uptake area of Hospital A. In Table 1, we describe eligible patients and reasons for nonparticipation.

### Data Collection and Analysis

The data included both qualitative and quantitative data from semistructured interviews and the READHY questionnaire. The 25 participants were asked to fill in the READHY questionnaire, followed by individual interviews with the first author (RT). The interviews were performed at the hospital to gain an understanding of the experiences, competencies, and attitudes of older patients toward the use of ICT and their management of nutritional needs. An interview guide based on the dimensions from the READHY tool was developed and used. The first author undertook the data collection. We planned to include 10-12 participants from each hospital unit and to evaluate whether categories of participants scoring high and low in the READHY themes of self-management, social support, and eHealth literacy were represented, and that saturation with respect to new aspects of ICT usage or understanding of nutrition was achieved. For Hospital B, we lacked some male representatives and therefore included a total of 13 participants from this hospital.

Qualitative content analysis was used [27,28]. To ensure trustworthiness, the analysis and interpretation of the qualitative data were carried out as follows. The coding of the first three transcribed interviews was reviewed and discussed with all authors. The transverse analysis and interpretation were performed in collaboration between two authors (RT and TL) and were discussed with the other author (LK) until consensus was reached. The interviews were conducted, transcribed, and analyzed in Danish. Quotations included herein were translated into English by a bilingual translator in collaboration with RT to ensure the meaning was not distorted.

### Theoretical Framework

As previously reported, we used the READHY instrument as a theoretical framework to explore the informants’ capacity to utilize an eHealth solution. READHY is a psychometrically validated instrument developed to measure an individual’s health technology readiness [24]. It consists of 65 items covering 13 dimensions from three distinct instruments measuring the concepts of eHealth literacy, health literacy, and self-management. The READHY instrument is based on the concepts of eHealth literacy comprising the seven dimensions from the eHealth Literacy Questionnaire (eHLQ) [29], which address: (1) the user’s knowledge and skills (eHLQ1, eHLQ2, and eHLQ3); (2) the user experience (eHLQ6 and eHLQ7); (3) the users’ trust toward digital technology (eHLQ4); and (4) the user’s motivation for engaging with the technology (eHLQ5). It has been argued that an individual’s capability to utilize eHealth is influenced by their competence in managing the burden of treatment and illness, as well as the social context such as social support [23,24]. READHY addresses social aspects such as support from relatives and health care professionals in two dimensions from the Health Literacy Questionnaire (HLQ; HLQ1 and HLQ4) [30]. Additionally, READHY contains four dimensions from the Health Education Impact Questionnaire (heiQ) [31], which addresses perspectives of self-management: self-monitoring and insight into their own health (heiQ3), constructive attitudes and approaches (heiQ4), skill and technique (heiQ5), and emotional distress (heiQ8). The 13 distinct dimensions captured in the READHY instrument are measured on a Likert scale with the following response categories: 1, strongly disagree; 2, disagree; 3, agree; and 4, strongly agree. Within each dimension, the items sum up to a composite score: 1 is the least desirable score and 4 is the most desirable score.

### Ethical Considerations

Mandated by the Danish Data Protection Agency, the study was approved by the Capital Region of Denmark (local record number HGH-2017-021). The Regional Ethical Committee (j.nr H-17006045) evaluated the study and found that ethical approval was not required. Verbal and written information about the study were provided to all participants by RT and they signed an informed consent form.

## Results

### Patient Characteristics

A total of 25 out of 85 eligible patients were included in this study. The median age was 81 years and 13 (52%) of the patients were women. Further patient characteristics are summarized in Table 2. The results in Table 2, except for those related to digital use, were previously reported [26].

**Table 2:** Participant characteristics.

Variables			Total sample (N=25)		Hospital A (n=12)		Hospital B (n=13)		*P* value^a^
Age (years), median (IQR)			81 (72-88)		82 (73-90)		81 (70-88)		.55
Sex (female), n (%)			13 (52)		5 (42)		8 (62)		.32
Civil status; living alone, n (%)			13 (52)		6 (50)		7 (54)		.85
Digital use; use of ICT^b^, n (%)			16 (64)		9 (75)		7 (54)		.27
**School level, n (%)**									.40
	≤7 years	8 (32)		2 (17)		6 (46)			
	8-9 years	6 (24)		3 (25)		3 (23)			
	10-11 years	9 (36)		6 (50)		3 (23)			
	Upper Secondary School Leaving Examination	2 (8)		1 (8)		1 (7)			
**Education level, n (%)**									.25
	Comprehensive^c^	6 (24)		2 (17)		4 (31)			
	Short education^d^	11 (44)		4 (33)		7 (54)			
	Medium education^e^	6 (24)		4 (33)		2 (16)			
	Long education^f^	2 (8)		2 (17)		0 (0)			

^a^Pearson χ^2^ test was used for categorical variables and Mann-Whitney *U* test was used for continuous variables.

^b^ICT: information and communication technology.

^c^Corresponding to International Standard Classification of Education-2011 levels 1 and 2.

^d^Corresponding to International Standard Classification of Education-2011 levels 3, 4, and 5.

^e^Corresponding to International Standard Classification of Education-2011 level 6.

^f^Corresponding to International Standard Classification of Education-2011 levels 7 and 8.

### Quantitative Analysis

The informants were interviewed on the third day after admission. No significant differences in informants’ characteristics between Hospital A and Hospital B were found. The informants’ scores from the READHY instrument are summarized in Table 3. The informants from Hospital A had a higher score on 11 out of 13 scales. However, only significantly higher scores were found for two scales: “Self-monitoring and insight” and “Feeling understood and supported by health care providers.” Informants who used ICT had a significantly higher score than nonusers on 5 out of 7 scales within the eHealth literacy dimensions (Table 4).

**Table 3:** Readiness and Enablement Index for Health Technology (READHY) scores for the total sample and between patients from the two hospitals.

READHY dimensions^a^		Total sample (N=25), median (range)	Hospital A (n=12), median (range)	Hospital B (n=13), median (range)	*P* value^b^
**heiQ^c^**					
	heiQ3: self-monitoring and insight	2.8 (2.0-4.0)	3.2 (2.0-4.0)	2.7 (2.2-3.2)	.007
	heiQ4: constructive attitudes and approaches	3.2 (1.0-3.8)	3.2 (2.5-3.8)	3.2 (1.0-3.8)	.44
	heiQ5: skills and technique acquisition	3.0 (1.3-4.0)	3.0 (2.0-3.8)	2.8 (1.3-4.0)	.35
	heiQ8: emotional distress^d^	2.5 (1.2-3.5)	2.6 (1.2-3.5)	2.5 (1.8-3.5)	.51
**HLQ^e^**					
	HLQ1: feeling understood and supported by health care providers	3.0 (1.0-4.0)	3.8 (2.0-4.0)	2.8 (1.0-4.0)	.004
	HLQ4: social support for health	3.4 (1.0-4.0)	3.8 (2.2-4.0)	3.0 (1.0-4.0)	.14
**eHLQ^f^**					
	eHLQ1: ability to process information	1.8 (1.0-4.0)	1.9 (1.0-3.2)	1.8 (1.0-4.0)	.76
	eHLQ2: understanding of health concepts and language	2.8 (1.0-3.6)	3.0 (2.4-3.6)	2.6 (1.0-3.6)	.054
	eHLQ3: ability to actively engage with digital services	2.0 (1.0-3.4)	1.9 (1.0-3.2)	2.2 (1.0-3.4)	.79
	eHLQ4: feel safe and in control	3.0 (1.8-4.0)	3.0 (2.2-4.0)	2.8 (1.8-3.2)	.07
	eHLQ5: motivated to engage with digital services	2.4 (1.0-3.6)	2.5 (1.0-3.0)	1.8 (1.0-3.6)	.25
	eHLQ6: access to digital services that work	2.0 (1.0-3.0)	2.5 (1.0-3.0)	2.0 (1.3-2.8)	.78
	eHLQ7: digital services that suit individual needs	1.5 (1.0-3.3)	1.6 (1.0-3.3)	1.5 (1.0-3.0)	.68

^a^The dimension scores are based on following response categories: 1, strongly disagree; 2, disagree; 3, agree; and 4, strongly agree. A high score is a more desirable trait. The heiQ3, heiQ4, heiQ5, heiQ8, HLQ1, HLQ4, and eHLQ2 scores have been reported previously [26].

^b^Mann-Whitney *U* test.

^c^heiQ: Health Education Impact Questionnaire.

^d^Reverse score; a high score means a low level of distress.

^e^HLQ: Health Literacy Questionnaire.

^f^eHLQ: eHealth Literacy Questionnaire.

**Table 4:** Readiness and Enablement Index for Health Technology (READHY) scores for information and communications technology (ICT) users versus nonusers.

READHY dimensions^a^			ICT users (n=16), median (range)		ICT nonusers (n=9), median (range)	*P* value^b^
**heiQ^c^**						
	heiQ3: self-monitoring and insight	2.9 (2.0-3.7)		2.8 (2.2-4.0)		.95
	heiQ4: constructive attitudes and approaches	3.0 (1.0-3.8)		3.2 (2.6-3.8)		.33
	heiQ5: skills and technique acquisition	3.0 (1.3-4.0)		3.0 (2.0-3.8)		.49
	heiQ8: emotional distress^d^	2.5 (1.2-3.3)		3.2 (1.8-3.5)		.20
**HLQ^e^**						
	HLQ1: feeling understood and supported by health care providers	3.3 (1.0-4.0)		3.0 (1.8-4.0)		.84
	HLQ4: social support for health	3.1 (1.0-4.0)		3.6 (2.4-4.0)		.30
**eHLQ^f^**						
	eHLQ1: ability to process information	2.4 (1.0-4.0)		1.2 (1.0-1.8)		.004
	eHLQ2: understanding of health concepts and language	2.8 (1.0-3.4)		3.0 (2.2-3.6)		.09
	eHLQ3: ability to actively engage with digital services	2.5 (1.0-3.4)		1.4 (1.0-1.6)		<.001
	eHLQ4: feel safe and in control	2.9 (1.8-3.6)		3.0 (2.0-4.0)		.69
	eHLQ5: motivated to engage with digital services	2.7 (1.0-3.6)		1.8 (1.0-2.4)		.02
	eHLQ6: access to digital services that work	2.7 (1.0-3.0)		1.5 (1.3-2.0)		.02
	eHLQ7: digital services that suit individual needs	2.0 (1.0-3.3)		1.0 (1.0-2.0)		.01

^a^The dimension scores are based on following response categories: 1, strongly disagree; 2, disagree; 3, agree; and 4, strongly agree. A high score is a more desirable trait. The heiQ3, heiQ4, heiQ5, heiQ8, HLQ1, HLQ4, and eHLQ2 scores have been reported previously [26].

^b^Mann-Whitney *U* test.

^c^heiQ: Health Education Impact Questionnaire.

^d^Reverse score; a high score means a low level of distress.

^e^HLQ: Health Literacy Questionnaire.

^f^eHLQ: eHealth Literacy Questionnaire.

### Qualitative Analysis

#### Main Themes

**Figure 1:**
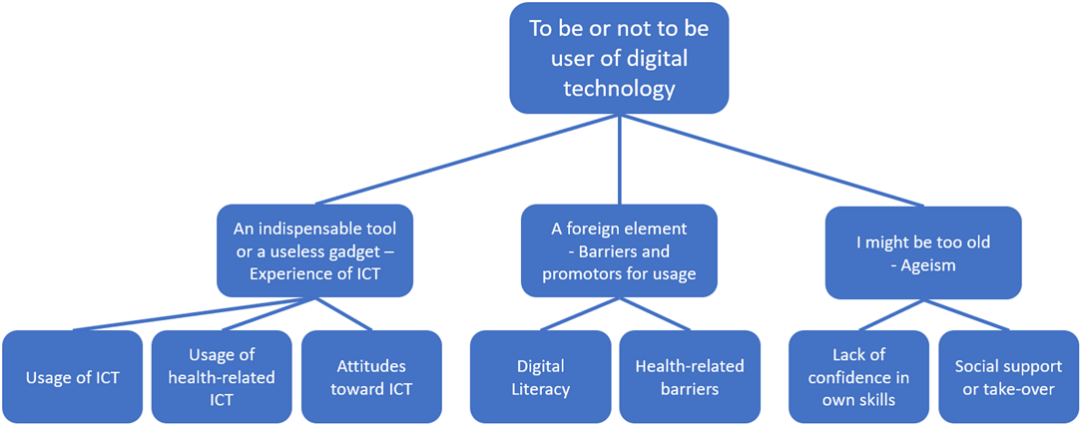
Main theme, subthemes, and subordinate themes. ICT: information and communication technology

From the qualitative analysis, one main theme emerged: To be or not to be a user of technology. There were three subthemes identified: (1) An indispensable tool or a useless gadget: experiences of ICT; (2) A foreign element: barriers and promotors for usage; and (3) I might be too old: ageism (Figure 1). The qualitative findings showed a noteworthy diversity in the informants’ attitude, use, and experience with ICT. The findings revealed how the use and nonuse of ICT was related to the informants’ expectations of derived benefits and their own competence.

#### An Indispensable Tool or a Useless Gadget: Experience of ICT

##### Theme Overview

This theme covers the diversity of the informants in their experiences and attitudes toward ICT. The informants’ experiences of ICT spread over a spectrum. One side of the spectrum included patients that used ICT on a daily basis and experienced it as an indispensable tool in their lives. The other side of the spectrum included informants who never used ICT and regarded it as irrelevant; some found it intimidating and some even considered it to be a threat to their usual way of living. In general, the nonuse of ICT was not a sign of rejection by the informants, but rather an expression of them feeling that they were not a target group for this technology.

##### Usage of ICT

Most of the informants used ICT at home on a daily basis, and several had various computer devices, including a personal computer, tablet, and smartphone. They used ICT for different purposes such as information seeking, communication with friends and family via email, managing finances, and entertainment. Use of social media such as Facebook was also mentioned. Notably, many of the nonusers of ICT had been introduced to ICT earlier in life, such as through personal computer training in the local residents club or seniors club. However, the skills acquired at such training events had been forgotten, despite their initial interest:

I am member of a senior citizens club through HK (a trade union)…Yes, it is more than 10 years ago we got the chance to try a computer, and it was quite exciting.

Informant A; 87 years

When asked directly, the informant could not explain why she was not using ICT currently, except that she was managing just fine without it. The informants most frequently explained their nonuse of ICT as lack of need or interest. When asked if they would like to learn to use ICT, one informant responded:

Well, um, in a way, yes, but on the other hand: what would I use it for?”

Informant B; 81 years

##### Usage of Health-Related ICT

Use of ICT in relation to health and well-being was common, primarily to look up health information. The search engine Google was used by many, but others also mentioned the national health portal (sundhed.dk), which after logging in with a national personal identifier provides access to various health services, including the electronic health record, prescribed drugs, and paraclinical data. This portal also provides information about health services and resources, and on different conditions and how they are treated using a so-called patient “handbook” without needing to log in. The informants were mainly searching for information on diseases, treatments, and medicine. Beyond information seeking, some informants mentioned how they used digital services of their general practitioners (GPs) for booking appointments or renewal of drug prescriptions. The informants also used access to their electronic health record for information about their treatment. In general, the informants had limited experience with using ICT for monitoring their health conditions. One exception was a patient who used an app on his smartphone for monitoring physical activity (ie, the distance moved in a day). This informant differed from the others as he was younger. Health-related use of ICT was mainly focused on treatment and prevention of complications of an existing disease and, to a limited extent, on health promotion.

Daily use of ICT did not always encompass purposes related to health and well-being. For instance, several informants explained that they did not take advantage of the digital health services offered by the GP. This was not due to worries about digital safety. In general, ICT users trusted the security in the digital systems when sharing their data, and data security did not seem to be a concern among nonusers. For some informants, the use of health-related ICT was perceived as a risk of being a substitute for personal contact with the health care professionals (eg, their GP). Several informants explained how the information was generally better and more easily understood when received in person, and some expressed concerns about misunderstandings. Other reasons mentioned for not using ICT for health-related purposes was lack of knowledge, user competence, and interest. The latter was often an expression of lack of knowledge of the opportunities made available by the technology.

##### Attitudes Toward ICT

In general, the ICT users had a positive attitude toward ICT. Their narratives revealed how their attitudes were associated with their experiences of various ICT benefits in their everyday lives. Access to all kinds of information on the internet was especially appreciated:

I basically find a computer an indispensable tool. If you want to know something, well, ask the computer.

Informant C; 91 years

Some informants expressed how ICT helped them manage the challenges of living with a chronic condition, such as by providing information about illness and treatment. Easy access to information on the internet helped prepare them for more qualified conversations with health care professionals:

...It probably means that you are better prepared for at least some of the doctor’s consultations…I mean, in reality, it is all about asking the right questions.

Informant D; 73 years

Other informants described how using the GP’s digital services made appointment booking and renewing prescriptions easier, and therefore making interactions less dependent on the GP’s telephone hours. The analysis further revealed examples as to how ICT had a positive influence on compliance with medication, such as the timely ordering of medication by digital renewal of prescriptions and correct administration of medication due to easy access to information.

Not all informants considered ICT to be an indispensable tool. In general, the nonusers lacked interest in using ICT, as they did not consider it relevant. A negative attitude was not common, but was observed. One informant rejected digital communication from public authorities but still used ICT for email with friends and family.

But now, when you are being pushed, I feel genuinely annoyed over…um…digital pressure from society, from the municipalities. I feel it isn’t right (…) I have applied to be, what do they call it, not-digitalized, and I got approved

Informant E; 88 years

ICT was experienced as something new and unfamiliar, influencing their attitudes toward using it. For some, this attitude was a barrier for using ICT, whereas others embraced this challenge and embarked on learning new skills to overcome the difficulties.

I want to learn, he (son) shouldn’t tell me what to do, he should be teaching me how to, so I can do it myself; otherwise I will have a gigantic problem on my hands as soon as he is out of the door

Informant F; 76 years

Despite the challenges experienced, these were not always a hindrance to using the technology. Generally, the informants accepted the occasional challenges and the fact that they sometimes needed assistance with completing the task they were engaged in. Technical challenges such as an inaccessible system or difficulties operating the system were met with patience and confidence. An acceptance attitude was apparent, acknowledging that things may take time and it was sometimes a matter of waiting, either for the system to work again or for the necessary support to be available.

But sometimes it’s a real mess (laughing).

RT: What is it that’s a mess?

It’s all of it, isn’t it? I mean, (…) then I wait a bit, then I try again (…) then it usually ends up working

Informant G; 70 years

It became apparent in the informants’ narratives that the nonuse of ICT could not necessarily be explained by being technology-averse in general, as some of the nonusers handled other technological devices without problems, such as for monitoring their blood sugar.

#### A Foreign Element: Barriers and Promotors for Usage

##### Theme Overview

Personal attributes such as health-related challenges and limited digital literacy among the informants were barriers for their use of ICT. The informants generally indicated an acceptance of the barriers experienced, and they acknowledged that they often depend on support that is mainly provided by their children.

##### Digital Literacy

A consistent theme was that the informants felt unfamiliar with the language and concepts of the digital systems and had a hard time understanding them. Some emphasized that this was not due to cognitive limitations, as they felt they had good linguistic skills, but rather to their introduction to ICT late in life:

It’s not like I am linguistically challenged but there have been some instructions where I was thinking: what in the world are you talking about?

Informant H; 81 years

The informants mentioned examples of how they encountered new words that made no sense to them, which complicated navigating the system. Age was often considered the prime reason for these linguistic challenges. The informants were older, and technology had entailed estranged procedures and language for which they had no prior experiences to cope with. Time was experienced as passing fast, particularly with regard to the digital age, introducing swift changes in functions as well as language and expressions in relation to technology.

Because when I was 18-20 years old, nobody said anything about digital files, we didn’t say “stand-by” either, we said “stop.” (…) there are so many new words and things in the systems, and you can’t keep up, also because time passes so quickly for us

Informant C; 91 years

One informant used ICT to stay in touch with friends and family by email, but she found it challenging, as she sometimes forgot which button to press. This informant labeled herself as suffering from technological illiteracy.

##### Health-Related Barriers

Various health-related barriers such as arthritis in the fingers or reduced vision were described as making it difficult to operate certain devices, including when using touchscreens on tablets and smartphones. Informants with impaired vision experienced the use of tablets and phones with small screens challenging. Many preferred the computer as it provides a larger screen. Previously, some of the nonusers had used a personal computer, but had experienced increasing problems over time, which they related to a decline in their cognitive skills such as difficulties with learning and memory. Thus, the informants experienced challenges making the use of both hardware and software either difficult or impossible. Mostly, the obstacles experienced using ICT were related to personal barriers and not to a lack of functionality of the ICT systems.

But then they introduced new systems, and I have a Windows10, which for me is more complicated. And so, I find it harder to learn now. (…) there is no doubt I am having a hard time figuring things out. This is also because I cannot see things properly. It is a terrible show-stopper that I cannot see properly. This is my biggest challenge.

Informant I; 93 years

However, one informant attributed the challenges to the digital system. He was an experienced ICT user and differed from the other informants as he was younger:

You can say, they are different systems … iPad and iPhone are different from PC, right? It doesn’t always work well together.

Informant J; 69 years

#### I Might Be Too Old: Ageism

##### Theme Overview

This theme describes an understanding that appeared to be common both among the informants and also the social network, indicating that increased age was associated with limited competence to benefit from ICT usage. This understanding seemed in itself to be a substantial barrier for not using ICT at all but also prevented ICT users from expanding their use to health-related purposes.

##### Lack of Confidence in Own Skills

A general lack of confidence in their own skills in ICT use among the informants was apparent throughout the data both among users and nonusers. This was often based on the attitude that age had the upper hand and made it increasingly difficult to use ICT. For some, however, this attitude was based on prejudice and not from real experience with ICT:

I am not so good at this sort of thing, and then I’d rather not do it at all (…) I keep telling myself I can’t and then I’d rather not.

Informant K; 89 years

Age was the dominant reason given by nonusers of ICT, combined with the assumption that the effort demanded to acquire the necessary skills was too great, and, in view of their remaining years, not worthwhile, particularly since many had no expectations for ICT to benefit them in their present situation and age. Even informants who actually used ICT lacked confidence in their possibilities in acquiring the necessary skills for using ICT for health-managing purposes.

I really don’t have the capacity or skills for such stuff, no, I can’t do that.

RT: But you are using that PC, aren’t you?

Yes, but not for that sort of thing, I mostly use it for fun

Informant L; 92 years

Apparently, the informants’ relatives (eg, the children) also assumed that the older patients were not able to benefit from ICT and that the way they use it may cause malfunction of the technology due to their lack of skills.

"Stay away from that (the computer) (…) You don't understand it anyways,” she says (the daughter)...She might be right! (…) But I’m told it’s not so hard. Although my children say: “You don’t need a mobile phone, you don’t understand it, anyway."

Informant M; 91 years

##### Social Support or Take-Over

The need for assistance with a variety of challenges that arose with using ICT was common. Generally, the informants experienced receiving the support they needed. Several described the various options for free technology support in their local resident community center or seniors club. Children and grandchildren were described as the main source of support, and very competent.

We have such great grandchildren who know much more than everybody else (laughs). That’s when we get to learn, right? And then I have a son-in-law who is an IT expert.

Informant N; 85 years

The informants expressed gratitude for the support from their children, but there were occasions where this support led to the children taking over instead. When asked if they would like to try the computer, one informant responded:

No (…) I’m just fine without. And if I needed help with anything, one of my children would do it for me.

Informant O; 79 years

Some informants had previously been introduced to ICT but had either stopped using it or never really started. This lack of use was seemingly not out of rejection of ICT, but more a passive decision fueled by the lack of expectations from their surroundings and the lack of confidence progressing steadily with age.

## Discussion

### Principal Findings

The aim of this study was to explore older patients’ competencies, preferences, and attitudes toward use of ICT, and to gain an understanding of both the barriers and facilitators for their motivation to engage with eHealth. Our findings contradict the perception that older patients cannot or will not use ICT. The qualitative and quantitative data revealed that older patients were indeed users of ICT, but their competence, ability, and preferences may differ from those of younger people. A main finding of this study was the large diversity in the informants’ experiences with the use of ICT. This spanned from daily use to no use at all. The majority of the informants used ICT on a daily basis, which was in alignment with former studies [15,21,32] as well as with data from Statistics Denmark, showing that 51% of the 65-74 year olds and 26% of the 75-89 year olds use the internet on a daily basis [20]. Several informants had experiences with health-related ICT use. Information seeking was common, but they had limited experience with monitoring their own health through ICT. However, this study also revealed that some informants never used ICT and were not motivated to begin.

The wide range in the use of ICT was also reflected in the differing competencies among the informants, indicating differing needs for support to utilize eHealth. A predominant factor for the informants’ user skills related to ICT was their age, as they had been introduced to it late in life. The qualitative data illustrated how many were not familiar with the “digital language,” and some experienced this as a challenge to be overcome, while others saw it as a barrier preventing them from actual use of ICT. This qualitative finding was also reflected in the low READHY score on the scale within the dimension “Ability to process information” (eHLQ1), which covers the capability to read, apply, and understand context-specific language such as health and information technology [29]. In contrast, there was no difference in scores between users and nonusers of ICT on the scale within the eHealth literacy dimension “Understanding of health concepts and language” (eHLQ2), which covers the feeling of having knowledge of basic physiological functions and how to take care of one’s own health [29]. Furthermore, we found no difference between the scores of users and nonusers on the scales within the four self-management dimensions (heiQ3, heiQ4, heiQ5, and heiQ8), which indicates that the nonusers’ readiness to engage with ICT was limited by a low level of eHealth literacy rather than by their health-related self-management competence. In a hospital setting, it is expected that older patients with acute illness are even more challenged in their ability to obtain and understand information. This emphasizes the importance of providing older patients with support for computer skills and introducing technology in a language familiar to them.

The informants using ICT differed from the nonusers by having experience with the benefits of ICT. This experience of ICT as a useful tool for everyday tasks seemed to have a facilitating influence on ICT usage, which corresponds with several other studies [15,16,21,33]. Both de Veer et al [15] and Van Houwelingen et al [21] found that acceptance and use of ICT were influenced by trust in its derived benefits, also termed “performance expectancy” in technology acceptance theory. A prerequisite for assessing the potential benefits of using ICT, including eHealth, is first and foremost knowledge of the possible assistance and support it provides. Many of our informants lacking interest in ICT were not aware of its potential to help their health. Seemingly, a main reason for not using ICT was lack of knowledge of the beneficial use rather than rejection. Other authors have argued that older patients will use technology if they perceive it as useful [16]. These findings emphasize that health care professionals have an important role in promoting the benefits of using eHealth. In relation to the educative intervention we are going to develop, it is essential to provide older patients with knowledge of how this specific nutritional eHealth solution will enable them to eat sufficiently, and most importantly how sufficient food intake will have a positive effect on their health and well-being.

A prevailing finding was the informants’ lack of confidence in their own competence in using ICT, and how it affected their usage. Theoretically, lack of confidence in one’s own competence relates to the concept of self-efficacy, which is defined as “people’s beliefs in their capabilities to produce given attainments” [34]. Self-efficacy influences individuals’ health behavior intentions, and in this case engagement with an eHealth solution [34,35]. The informants’ low score within the READHY dimension “Ability to actively engage with digital services” (eHLQ3) [29] supports the qualitative finding of low confidence in using ICT. The nonusers’ score was significantly lower than that of the ICT users, and was also lower compared with that reported in other studies using the same instrument [36,37]. Several other studies have found that older patients’ level of self-efficacy influences their use of eHealth. In a Dutch survey study (N=1014), de Veer et al [15] reported self-efficacy to be significantly correlated to older patients’ intention to use eHealth applications. In another study based on data from a questionnaire (N=256) and interviews (N=15), Van Houwelingen et al [21] reported that self-efficacy predicted older patients’ effort expectancy (ie, their belief in how hard or easy it is to use the technology), which was positively associated with their intention to use telehealth.

In future interventions, when introducing older patients to eHealth, it will be important to be aware of and increase their self-efficacy with use of technology. According to social cognitive theory, an individual’s self-efficacy can be improved through mastery experience [34]. Therefore, a key factor in motivating older patients to engage with eHealth is to introduce it in a way that they can perceive the technology as both useful and manageable. Thus, in a hospital setting, when introducing eHealth, it is crucial to provide older patients with sufficient technical support to make them feel confident in using eHealth.

The social context such as feeling understood and having the necessary support from relatives and health care professionals influences an individual’s capability to utilize eHealth [24]. The informants in our study experienced having the necessary support, including technology support from their relatives, in most cases their adult children. Moreover, they generally felt understood and supported by the health care professionals. The above qualitative findings were also reflected in the results from READHY scores, as the total sample had a high median score (above 3) on scales within the dimensions measuring their feelings of being understood and supported by health care professionals and their relatives (HLQ1 and HLQ4). It is noteworthy that the informants with a median age of 81 years had scores in the above-mentioned two scales similar to those reported in the Danish validation study covering the general population with a mean age of 53 years [38].

A lower level of health literacy among older patients has been reported [39]. This study indicated that older patients, even those with acute and chronic illness, often have health literacy resources in terms of support from their social network and trust in the health care system. However, it seems that these resources may not enable or motivate engagement with ICT. As described above, the informants lacked knowledge of the possibilities and benefits of using eHealth, despite their frequent contact with the health care system. Hence, these patients were seemingly not informed and motivated to use ICT for health-related purposes by the health care professionals they met. This may be explained by a general perception of health care professionals that older patients are not motivated for and able to utilize eHealth [13,14]. Paradoxically, the social network appeared for some to become an obstacle to the use of ICT. In accordance with other studies [15,16], we found that helpful relatives risked taking over the tasks and thus reduced the older person’s need to use ICT. Furthermore, the informants’ lack of confidence in their own ICT competence was also shared by their relatives. A prevailing theme in the qualitative data was ageism, defined as “the stereotyping, prejudice, and discrimination against people on the basis of their age” [40]. The informants’ perception that they, due to their high age, lacked ICT competence was in some cases confirmed by their relatives. Nevertheless, in accordance with other studies [16,21], this study showed how the informants valued the support and guidance from relatives, indicating that it is important to involve relatives when introducing eHealth to older patients. The relatives must perceive the older patients to potentially be capable of using and benefiting from eHealth. Subsequently, the educative intervention must target both patients and relatives.

An important finding in this study was the informants’ perception of ICT usage leading to less personal contact with health care professionals. Consistent with other studies [33,41], the informants in our study preferred personal contact when communicating with health care professionals. The nonuse of health-related ICT was neither due to mistrust in security nor sharing data in digital systems but rather to the perception of digital communication detracting from the personal interaction with the health care professionals. Thus, older patients should be introduced to eHealth as a tool adjunct to the personal guidance and feedback from the health care professionals, enabling them to participate in their own health care. Moreover, we found that older patients may have some preferences for choice of computer devices due to health-related barriers (eg, a bigger screen due to reduced vision or a computer with a keyboard instead of a tablet due to obstacles with touch). These aspects must be considered when planning the implementation of eHealth solutions in a hospital setting to ensure older patients’ successful involvement.

We found demographic differences in the samples from the two hospitals (ie, lower educational level), corresponding with differences in their READHY scores. In accordance with other studies [41], this underlines that patients with a lower educational level may need more and individualized support to utilize eHealth.

### Strengths and Limitations

One important strength of this study is that the themes appeared across the sample regardless of differences in gender, age, and socioeconomic background. The sample size was small, but nevertheless heterogeneous in terms of the older patients’ gender, age, use of ICT, and educational attainment. In a small sample, heterogeneity may add strength as a pattern across variation highlights central aspects of the phenomenon [42]. Another important strength was the use of READHY as a theoretical framework, which ensured that we captured relevant perspectives in relation to competence for ICT usage. The use of a qualitative design allowed for additional perspectives to emerge. By combining the qualitative and quantitative results, we achieved a nuanced understanding of this group of patients. Furthermore, READHY is a multidimensional instrument encompassing the many aspects influencing individuals’ abilities to engage with eHealth, and allows for gaining a broader understanding of older patients’ resources and barriers to be addressed in an educative intervention.

This study also has some limitations. The sample consisted of 25 patients, and 60 of the 85 eligible patients were excluded due to cognitive impairment, either permanent or acute, which negatively affects the transferability of the findings. Furthermore, the informants’ narratives might have been affected by their situation when they were interviewed (ie, being acutely ill and hospitalized).

### Conclusions

This study indicates that a large group of older patients are potential users of ICT, but their usage showed wide variation, which was also reflected in their competencies, preferences, and attitudes toward the use of ICT. This group of patients has competencies and resources related to self-management and social support that should be utilized when introducing them to eHealth in a hospital setting. An important facilitator for motivating older patients to engage with eHealth is knowledge of the benefits derived from eHealth, and how this may assist them in managing health-related challenges. When introducing health technology to patients, health care professionals should be aware of how both their own assumptions and attitudes and those of relatives may cause a barrier, as well as an insufficient level of patients’ knowledge, skills, motivation, and confidence.

## Abbreviations

eHLQ: eHealth Literacy Questionnaire
GP: general practitioner
heiQ: Health Education Impact Questionnaire
HLQ: Health Literacy Questionnaire
ICT: information and communication technology
READHY: Readiness and Enablement Index for Health Technology
